# Enablers and barriers to introduction of obstetrics ultrasound service at primary care facilities in a resource-limited setting: a qualitative study in four regions of Ethiopia

**DOI:** 10.1186/s12884-022-04609-y

**Published:** 2022-04-02

**Authors:** Meselech Assegid Roro, Abebech Demissie Aredo, Tesfaye Kebede, Abiy Seifu Estifanos

**Affiliations:** 1 Department of Reproductive, Family and Population Health, School of Public Health, Addis Ababa University, Addis Ababa, Ethiopia; 2 Department of Radiology, School of Medicine, Addis Ababa University, Addis Ababa, Ethiopia

**Keywords:** Limited obstetrics ultrasound, Vscan ultrasound, Enablers, Barriers, Qualitative

## Abstract

**Background:**

The World Health Organization **(**WHO) recommends a minimum of eight ANC contacts during pregnancy, of which having one ultrasound examination before 24 weeks of gestation is indicated. Ultrasound plays a significant role in the surveillance and management of high-risk pregnancies. However, the obstetric ultrasound coverage in resource limited settings remains low. Evidence examining the barriers and facilitators to obstetrics ultrasound use in a resource-limited setting like Ethiopia is lacking. This qualitative study explored the facilitators and barriers to introducing obstetric Vscan Access ultrasound in primary health care facilities in Ethiopia.

**Methods:**

The study employed a qualitative descriptive exploratory study design using in-depth interviews (IDIs) and focus group discussions (FGDs). The study participant were mothers who have had recent birth, community members, maternal and newborn service providers, and their managers. We employed an inductive thematic analysis to analyze the data.

**Result:**

We conducted a total of ten FGDs, three with community members and seven with maternal and newborn service providers, and 52 IDIs with the service providers and health facility managers. Two major themes, health system related and client-related factors, emerged from the analysis. The health system related enablers include increased knowledge and skill of the providers, improved mothers and providers’ motivation, increased service utilization, and improved quality of maternal and newborn care (MNC), and enhanced referral system. The health system related barriers include service interruption, staff shortage/workload, and the providers’ limited capacity. Under the main theme of client-related factors, barriers include perceived limited knowledge and skills of providers and the small size of the ultrasound machine while the facilitators include mothers’ needs and interest in ultrasound scan, availability of free of charge ultrasound service, and increased demand for ultrasound scan service.

**Conclusion:**

Our data suggest that the health system provides an enabling context to introduce limited obstetric ultrasound service and routinely provide the service through mid-level maternal care providers at primary health care level in resource limited settings. Overcoming the health system and client related barriers will maximize and sustain the use of the technology.

## Background

Complications related to pregnancy and childbirth are the leading causes of maternal mortality worldwide. Of the estimated 295,000 maternal deaths worldwide, more than 99% of occur in low and middle-income countries [1]. Ethiopia is among the top 15 countries contributing to the high burden of maternal deaths. Evidences show that the implementation of high-quality, evidence-based interventions can prevent the majority of these deaths.

Antenatal care (ANC) is known to improve pregnancy outcomes and reduce maternal and infant morbidity and mortality [2]. It also provides a unique opportunity for screening and diagnosis, health promotion, and disease prevention among pregnant women and communities [3]. As an entry point to the health care system for pregnant women and their families, ANC increases the likelihood of assistance by a skilled birth attendant [4]. The revised World Health Organizations (WHO) guideline recommends a minimum of eight ANC contacts during pregnancy, including one ultrasound examination before 24 weeks of gestation.

Ultrasound scan is useful to estimate gestational age, detect multiple pregnancies, placental location, fetal well-being, and fetal anomalies [5]. It also plays a vital role in the surveillance and management of high-risk pregnancies, reducing the need for obstetric interventions and the risk of perinatal death [6]. Furthermore, availing routine obstetric ultrasound scan service at the lowest level of health service delivery points increases the likelihood of ANC attendance and motivates pregnant women to deliver at health facilities [7].

While obstetric ultrasound remains an integral component of prenatal care in high-resource settings [8], despite the gradual increase in access, most women in sub-Saharan Africa do not have access to an ultrasound examination [9]. In these settings, obstetricians, radiologists, and medical officers are primary providers of obstetric ultrasound service [10]. Lack of these high-level health professionals, inadequately trained providers [11], and shortage of ultrasound equipment and supplies limit availability of the ultrasound scan services [12, 13]. However, enabler and barriers to introduction of ultrasound scan services at lower level health facilities in resource limited settings are not adequately studied.

For settings with limited health system capacity, short courses on basic obstetric ultrasound for mid-level health professionals with no prior experience on ultrasound was found to effective [14], feasible, and efficient [15]. In Ethiopia, the Ministry of Health (MoH), in collaboration with the General Electric Health Care (GEHC) project, introduced limited obstetrics (Vscan Access portable) ultrasound machines in selected primary health care facilities. The intervention had three main components: training of midwives or nurses who provide maternal and newborn service at the hospital and health center level, providing Vscan Access ultrasound machine, and conducting post-training mentorship. Ethiopian midwife association and Ethiopian radiologist association provided the training and mentorship. GEHC donated the Vscan Access ultrasound machine. This qualitative study examined facilitators and barriers to introducing obstetric Vscan Access ultrasound in primary health care facilities in selected health facilities in Oromia and South Nation National People (SNNP) regions and Addis Ababa and Dire Dawa city administrations.

## Methods and materials

This study was part of the evaluation of the GEHC supported primary and referral care for mothers and babies collaboratively implemented by the MoH and Regional Health Bureaus (RHBs) in Addis Ababa, Dire Dawa, Oromia and SNNP.

### Study setting

We conducted the study in two of the nine regional states and two administrative cities of Ethiopia. The study areas were Alaba special woreda of SNNP region, East Shoa Zone/Adami Tulu woreda of Oromia region, Akaki Kality sub-city of Addis Ababa City Administration, and Sabian operation area of Dire Dawa City Administration. The MoH and the four RHBs purposively selected the intervention areas. We included primary healthcare facilities (primary hospitals and health centers associated with the primary hospitals) where the four regions’ RHBs implemented the study interventions.

### Study design, participants and sampling procedure

We employed a qualitative descriptive exploratory study design to explore and describe the experience of provision of ultrasound service at primary health care units by mid-level health care providers.

### Recruitment of the study participant

In the study, we included health facility managers, Maternal and Child (MCH) case team leads, maternal and newborn care providers, recent mothers (mothers who had a live birth within 3 months before the interview), and community members. We purposively selected study participants to capture a broader range of experiences of providers and users of the Vscan Access ultrasound services. We recruited the study participants from the 29 health facilities implementing the interventions: four primary hospitals and 25 health centers associated with the hospitals in the four regions’ urban and rural areas.

In each of the health facilities, our qualitative research assistants recruited the study participants with the help of the head of the hospital or health center for the in-depth interview (IDI) and focus group discussion (FGD). We conducted the FGDs with maternal and newborn health care providers (midwives or nurses) who work in the maternity care unit, and available during the data collection period and willing to participate in the discussion. Besides, we conducted FGDs with community members who have had experience in using maternal health services in the health facilities. For IDIs, we included health managers of the intervention hospitals and health centers and mothers who have had a live birth in the 3 months before data collection. Our interviewers identified the mothers with the help of the village leaders of respective areas.

### Data collection procedure

Five qualitative research assistants who have health or related professional background with master’s degree training, experience in qualitative data collection and management, and fluent in the languages spoken in the study areas collected the data. Prior to the actual data collection, the data collectors were trained on the objective, methods, and tools of the study for three days. We prepared semi-structured guides to facilitate the FGDs and IDIs [16]. The guides were developed in English and then translated to the languages spoken in the four regions (Afan Oromo for Oromia and Amharic for the rest of the regions). Before actual data collection, we conducted pretesting of the interview guides and procedures and held debriefing meetings with the qualitative research assistants. The pretesting helped to estimate the time required to complete the FGDs and IDIs, refine the guides, and address the research assistants’ questions and concerns about interviewing procedure and developing field notes.

Our qualitative research assistants invited health facility managers and MCH case team leaders at health facilities to participate in the IDIs. The research assistants interviewed the participants in the rooms with auditory and visual privacy after fully informing them about the study and obtaining their consent. Similarly, the research assistants conducted the IDI with the MNC providers in their usual workplace, voluntarily, and respecting visual and auditory privacy. The IDIs with health manager and MNC providers lasted between 30 to 90 min. The interview procedure for IDIs with mothers, who recently delivered in this selected study facilities, followed a similar consent process and were conducted inside their home or in their garden within their compound. The IDIs with the mothers lasted between 30 to 59 min.

Our research assistants conducted the FGDs with the MNC providers in offices next to the delivery room or in the providers’ duty rooms (the room where the health workers sleep during the night shift or use to change their clothes). The number of the FGDs’ participants varied from four to six, depending on MNC providers’ availability at the selected health facility. The FGDs lasted between 60 and 120 min.

The research assistants conducted the FGDs with community members in a central meeting spot that offers comfort and privacy. At the end of the FGDs, the community members received a small incentive (bars of soap) as compensation for their time. The size of the community FGDs participant varied from six to seven. The FGDs with the community members lasted between 30 and 60 min.

### Data analysis

We employed an inductive thematic analysis approach to analyze the qualitative data. The data analysis started in the field before the completion of the data collection. The qualitative research assistants took field notes during the interview and discussion. Immediately at the end of each of the IDIs or FGDs, research assistants developed expanded field notes after listening to the audio-records and enriching the notes they captured during the interview and discussion. ASE reviewed the expanded field notes and held debriefing meetings with the research assistants on crucial learnings gained from each IDI and FGD and areas to focus on in the subsequent interviews and discussions.

Our analysis steps followed reading of the text, coding, categorizing (reducing the data), and interpreting. ADA imported the expanded field notes into the NVivo version 12 data analysis software. After importing the expanded field notes into the software, ADA thoroughly read the entire text, highlighted and selected words or sentences and coded them under a phrase that explains the coded text. Then, after coding the entire data, ADA examined the codes to form categories and subcategories. Next, MAR and ASE reviewed the coded data, categories, and subcategories, which MAR and ADA further categorized into the major themes and sub-themes.

## Results

### Characteristics of the study participants

We conducted a total of ten FGDs, three with 19 community members and seven with 39 MNC providers. Additionally, we conducted 52 IDIs, 23 with health facility managers, six with recent mothers who have had a live birth within three months before the interview, and 23 with MNC service providers. All the recent mothers who participated in the FGD were married, 22-30 years old, about half of them attended above secondary school, about three-fourth of them had unskilled manual work as an occupation, and most of them had between two-three children. The MNC providers who participated in the FGD were 20-37 years old.

### Enablers and barriers of introducing obstetrics ultrasound

**Fig. 1:**
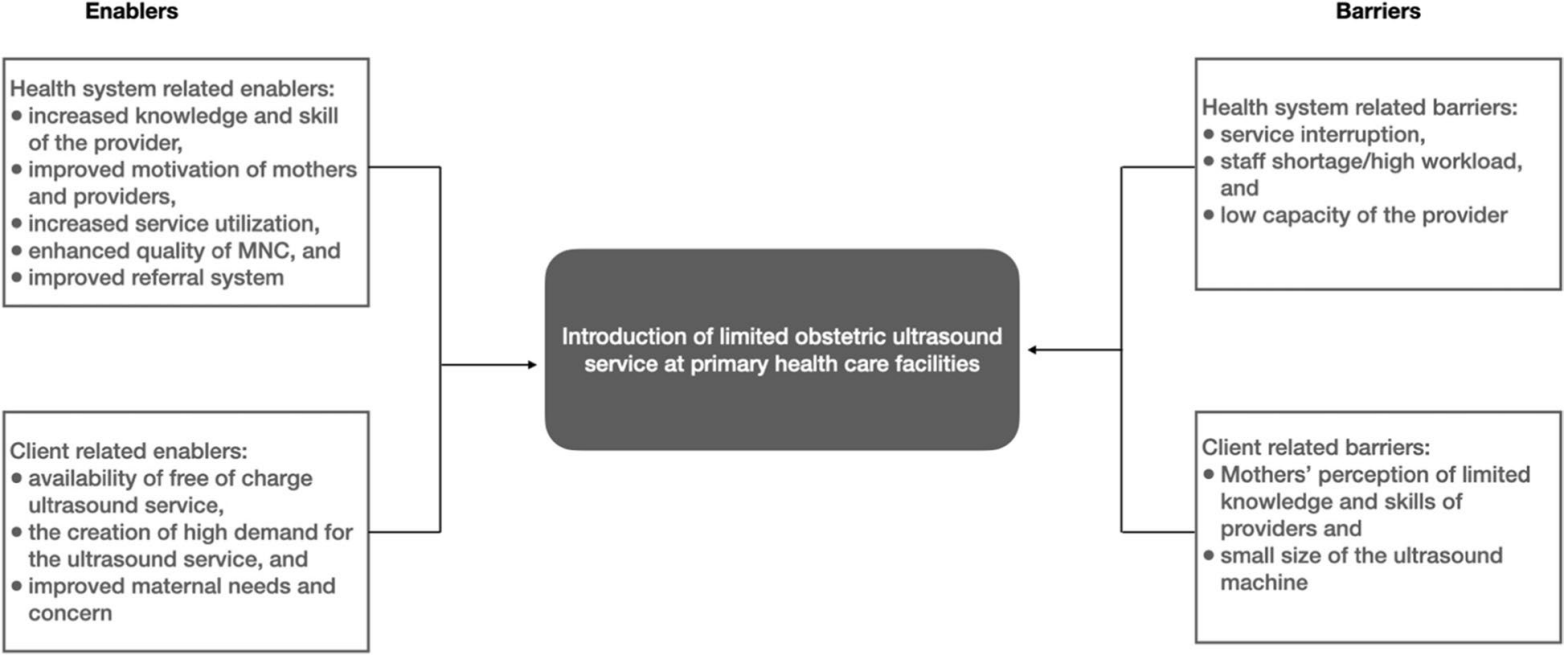
Enablers and barriers to introduction of obstetric ultrasound service at primary health care facilities, Ethiopia

Analysis of the IDIs and FGDs identified two major themes and 12 sub-themes related to enablers and barriers of introducing obstetrics ultrasound. The major themes identified were health system and client-related factors (Fig. 1).

### Health system-related enablers

#### Improved health workers knowledge and skills

MNC providers reported that the training provided to them on Vscan ultrasound as part of the introduction of the technology in their facilities built their knowledge and skill. They felt that the knowledge and skills they gained improved their confidence and helped them to better diagnose obstetric complications.

*"We received an ultrasound machine and training on how to use it. The training helps me in early identification of maternal and fetal complications. It makes me confident about what action to take in any situation." (MCH case team leader)*

#### Increased health workers and clients’ motivation

The health service managers noted that MNC providers were motivated and interested in providing the ultrasound examination for pregnant women because they were able to diagnose complications and take recommended action. It also attracted the community and created an opportunity to improve uptake of other health services provided by the health facilities. The maternal and newborn service provider asserted that the availability of the ultrasound service makes mothers happy and improves their interest to show up at their next scheduled ANC visit.

*“Availability of ultrasound scan is another reason for the increase of ANC uptake. Pregnant mothers who received the ultrasound service discuss the service with other pregnant mothers and then motivate them to come to this health center." (MCH case team leader)*

The MNC providers believe that the introductions of the Vscan ultrasound service at the health center level makes the service accessible to pregnant women and motivated them to come to the health facility to use the ANC service. According to the MNC provider, hearing their fetal heartbeat through the ultrasound machine increases the pregnant women’s satisfaction with their service and their trust in the MNC providers.

*"(The number of) ANC users increased from time to time, especially after the introduction of ultrasound. (FGD, MNC provider at a hospital)*

#### Increased maternal and newborn service utilization

The training on the ultrasound technology, the supportive supervision, and the Vscan Access ultrasound machine contributed to improved maternal and newborn service uptake in their health facilities. Some of the MNC providers think that the health facilities’ ultrasound service was the primary reason for increased delivery service uptake they observed.

*"When ultrasound service was not available, we used to send mothers to hospital for further diagnosis, but now this problem is solved. The ultrasound service (in our facility) increased the annual number of deliveries to about 300, from less than a hundred in the previous year". (Maternal newborn service provider)*

The dissemination of information about the availability of the service played a great role; mothers who didn’t use ANC service previously were motivated to use the service when they heard about the ultrasound service availability.

#### Improved quality of MNC

The participants believe that the availability of Vscan Access ultrasound at the primary health care level has highly contributed to improved quality of the MNC care they provide and their confidence in the service they provide and receive.

*"The presence of ultrasound increased our service quality and increased the interest of mothers to come to our health center to get the service." (Health center manager)*

*"There were different materials added to the facility that improved the quality of the service (the health center provides), for example, (the) ultrasound." (Mother who recently give birth at the health center)*

#### Improved referral system of the facility

Furthermore, the study participants reported that the Vscan Access ultrasound service improved the referral management at the health centers and primary hospitals because they were able to identify complications, make correct diagnosis, take the right decisions, and make the appropriate referral decision at the right time.

*“The (Vscan Access) ultrasound at our health center helped us to make correct diagnoses and referrals. (MNC provider at health center)*

*“The availability of equipment like ultrasound contributes much more to (improved) referral management.” (Health center manager)*

In addition, the Vscan Access ultrasound service helped MNC providers to avoid unnecessary referrals of pregnant women to higher level health facilities. Using the ultrasound machine, they were able to identify mothers who can or can’t deliver at the health center and need a timely referral to a higher level.

*"The Vscan Access the ultrasound reduces the referral of mothers (to higher level facilities) because we could manage different complication using the ultrasound." (MNC provider)*

### Health system-related barriers

#### Vscan access ultrasound related barriers

Interruption of the ultrasound service was a common challenge MNC providers frequently reported. According to the MNC providers, the ultrasound breakage is a major reason for discontinuity of the ultrasound service in their facilities. The maintenance of the ultrasound machine is done outside the health facility and takes long time, resulting in interruption of the service.

*"We used to have one (Vscan) ultrasound machine, but it is not functional now. We use a fetoscope to detect the fetal heartbeat, and the finding might differ from one midwife to the other. We might refer a mother for fetal distress because of poor diagnosis (with the fetoscope)." (Health center manager)*

In addition to machine breakage, another reason for the ultrasound service interruption study participants mentioned was the shortage of jell.

*"Sometimes we run out of jell for the ultrasound scan. During those times, we send clients to the nearby private or public hospital for an ultrasound scan." (MCH case team leader)*

In addition, shortage of electric power supply was discussed as an important barrier limiting the continuous provision of ultrasound scan services. Although some of the facilities have solar energy panels as an alternative energy source, they are not sufficient to accommodate the energy needs of different equipment the health facilities have.

#### Shortage of staff and high workload in the facility

Despite MNC providers’ interest in the Vscan ultrasound and their motivation to provide the service, their limited number affected the provision of the service to pregnant women. Availability of only one or two midwives per health center and few more at primary hospitals to manage all maternal and newborn health service mean they have high workload and do not prioritize provision of the ultrasound scan service, which is considered as additional care.

*"Shortage of human resources for maternal and newborn care is also another challenge we face in this health center. (The) only midwife (at our health center) provides family planning services, childbirth care service, antenatal care, and ultrasound examination everyday day……..." (MNC provider at health center)*

*"The ultrasound service is being given twice a week Tuesday and Friday after 8:00 o’clock local time (2:00pm)… We restricted the service to only two days per week because of the shortage of staff." (Health center manager)*

In addition to the shortage of midwives at the health center level, lack of access to training on Vscan Access ultrasound limited the service provision. Although the program trained two midwives from each facility on obstetrics ultrasound, some trained staff were transferred to other facilities without replacement, may not come to work due to social problems, maternity leave, or other reasons. As a result, the ultrasound service could not be routinely provided to the pregnant mothers.

*"We trained two staff (members) on antenatal (Vscan Access) ultrasound. One of them has left our health center. Sometimes, due to social or health problems, if she (the remaining staff) couldn't come, we can't provide the service. Rather, we appoint the pregnant mothers again and again for the ultrasound scan." (Health center manager)*

#### Low competence of the MNH providers

Limited capacity of the MNC care providers to provide the ultrasound scan was another barrier identified as a reason for poor performance of the ultrasound service provision. Some of the MNC providers stated that they received short training (ten days) without enough clinical practice, which left them with limited capacity to diagnose obstetric complication and low confidence to make a clinical decision, particularly on the first-trimester ultrasound scan.

*"The ultrasound training given to midwives is just a highlight. It is better if they are well-trained." (Health center manager)*

They feel that they have a skill gap to provide the service independently, and some of them believe that radiologists should provide the service. Despite the availability of providers trained on ultrasound scans in some health facilities, they continue to refer pregnant mothers to a private or public hospital for an ultrasound scan.

*"As to me, the availability of the ultrasound machine at health center level does not reduce the referral out due to the fear of death (maternal or neonatal), which means still they (the MNC providers) have a capacity gap." (MNC provider)*

### Client related enablers

#### Availability of free of charge ultrasound service

The provision of the ultrasound service free of charge was one of the important enabling factors that motivated the mothers to use the service. However, not all health centers provide the ultrasound services free of charge.

*"Last time I came across IUFD, and I have used the ultrasound to confirm and refer her to the hospital. The availability of ultrasound service free of charge saved the mother from the cost she would incur for the ultrasound (scan). When working in the ANC room, I used to give an ultrasound scan three times a week for five mothers per day. The (free) ultrasound service is vital for mothers who can't afford to pay for the service." (MNC provider)*

#### High demand for the ultrasound service

In general, pregnant women want more ultrasound examination than they received. Pregnant women showed a high interest in the ultrasound scan.

*"(All) mothers who visited our health center for antenatal care ask for the ultrasound service." (Health center manager)*

The provision of the ultrasound service at the primary care level increased mothers’ satisfaction and trust in the health care providers. Hearing fetal heartbeats and observing the fetal movement in the womb during the scan makes the mother happy and interested to show up at their scheduled ANC visits

*"Some mothers come, and they say 'I feel some pain, please examine me using the 'machine,' by machine they meant the ultrasound." (MNC provider)*

### Client related barriers

#### Maternal perceptions and concerns

Pregnant women who visit health facilities show a keen interest in ultrasound scans. They think that they should have the ultrasound scan for each complaint and during all of their ANC follow-up visits. They also feel that other examinations provided during the routine ANC follow up are not as important as the ultrasound scan.

*"They haven't sent me to other hospitals to be seen by a computer (ultrasound) because they said I have no problem. I haven't taken the computer examination on my own either, since they didn’t prescribe the scan to me." (A lactating mother who recently give birth at a health center)*

Mothers feel that health care providers discriminately provide the ultrasound scan to some pregnant women. Even if the ultrasound service exists in the health facility, they feel, providers don’t provide the service consistently to all mothers. They feel that the health providers selectively provide the ultrasound scan to the mothers they know or their relatives.

*"In case of the ultrasound, I think they selectively provide the service to the mothers they know. Otherwise, they will not (provide the service)." (Health development army, FGD participant)*

#### Perceived limited knowledge and skills of providers and small size of the ultrasound machine

The satisfaction of mothers with and trust in the ultrasound scan they received at the health center by the trained mid-level provider affects their use of the ultrasound service. Some mothers have doubts about mid-level healthcare providers’ knowledge and skill in providing the ultrasound service.

*"They checked me with ultrasound and said the baby is fine. And, they gave me medicine, but I couldn't get better; rather, I felt worse. Then I went to "X" private hospital for another ultrasound examination because I doubted (the scan I received at) the health center." (Lactating mother who recently give birth at the health center)*

Besides the providers’ capacity, the mothers also doubt the quality of the ultrasound machine used at the health center. They think that the size of the Vscan Access ultrasound machine used at the health center was very small. For the mothers, the small size ultrasound might not show anything about the fetus and the mother’s health condition. Even if some health centers provide the ultrasound examination for free, because of the size of the machine, some mothers prefer the ultrasound scans at private facilities.

*"The ultrasound (machine) is very small; the screen is like the size of my cell phone, and I don't think what they see through that screen enables them to know about anything. There is normal ultrasound in other facilities; with that ultrasound, you can see things very well as if you are watching television and the doctors interpret things for you. That way, you feel satisfied. But, the ultrasound in the health center, I don't think that is big enough to see something even for health workers. (Mother who recently give birth at the health center)*

## Discussion

This study gives an in-depth insight into the major health system and client-related enablers and barriers to the successful introduction of V-scan obstetrics ultrasound service at the primary health care level in Ethiopia’s four regions. Our study identified health system and client related factors that enable and limit the effective introduction of Vscan ultrasound service at the primary health care facility. The health system enablers were also the effects of the availability of the ultrasound service. Maternal and newborn healthcare providers and their managers alike reported that the limited ultrasound service enhanced the providers’ knowledge and skill, increased motivation of health workers and mothers to use the technology, increased maternal and newborn service utilization, improved quality of MNC, and enhanced referral management. The client-related enablers include high demand for the service and availability of the service free of charge. Whereas, Vscan ultrasound machine functionality related issues, shortage of human resources and increased workload, and low providers’ competence were health system related barriers that hinder the successful introduction of the limited obstetric ultrasound service. The client-related barriers include maternal perceptions and concerns, perceived low knowledge and skills of providers, and the small size of the limited obstetric ultrasound machine.

Factors like increase in confidence of mid-level providers, increase in percentage of women attending ANC and facility delivery, pregnant women’s high demand for ultrasound examinations, women’s satisfaction with receiving ultrasound examination during prenatal care were enablers documented in the literature [13, 17–22]. These are consistent with the findings in our study. In addition to these, our study identified perceived improved quality of MNC and enhanced referral management as enablers.

Barriers to introduction of obstetric ultrasound identified from the literatures search included lack of training, lack of equipment, ultrasound machine malfunction, lack of maintenance, cost of equipment, lack of skilled personnel, and negative attitude of parturient, long distances to service providers, heavy financial cost, insufficient skills of ultrasound operators, long waiting time, and unsatisfactory previous scan experience [4, 7, 23–26]. The barriers our study identified are in line with the existing evidence. However, the long distance to service, long waiting time, and negative attitude of the parturient did not emerge from analysis of our data. Whereas, the increased workload and the small size of the limited obstetric ultrasound machine were additional barriers our study identified.

Our study showed that high workload along with staff shortage were the main health system barriers for the use of limited obstetrics ultrasound. When providers who are trained were absent from work or transferred to other facility the ultrasound examination service was interrupted. A study from Rwanda similarly documented the insufficient number of physicians as a barrier to provision of obstetrics ultrasound [4]. Furthermore, with only few midwives available at primary care level adding ultrasound scan service on top of their MNC services provision responsibility could increases workload to the already overstretched providers. Provision of routine obstetric ultrasound service requires training of sufficient number of clinical staff in the Vscan ultrasound technology along with assigning additional midlevel MNC providers [27].

Our result indicates that though mothers have positive views about the ultrasound use and showed high interest in the obstetrics ultrasound exams, they perceived that small sized ultrasound machine doesn’t show everything and the professional are unable to see everything from the small ultrasound machine's screen. Since poor satisfaction with the previous scan was the main barriers for prenatal obstetrics ultrasound use [18], alongside improving the Vscan ultrasound technology to have larger screen, it is important to provide adequate pre-scan education to the mothers to address their concerns and correct their perception on the size the Vscan ultrasound.

Our study further showed that the training on Vscan ultrasound combined with post-training mentorship and provision of the Vscan ultrasound machine improved the quality of MNC service provided at primary health facilities. Evidence show that when adequate training materials and methods are used, short term training provides significant acquisition of knowledge and practical skill for health care providers in Low- and Middle-Income countries [19]. Consistent with this, our data showed that following the introduction of Vscan ultrasound services mid-level MNC providers were able to detect and manage the pregnancy related complications early during the ANC follow up. The improved knowledge and skill of the MNC providers gained through the training and mentorship have as well influenced their motivation and enhanced the trust and satisfaction of pregnant women who used the ANC service. As a result, the number of mothers who use the ANC and delivery service at the primary health care level increased, which was also reported by other studies [7]. As documented elsewhere [28, 29] the integration of routine ultrasound scan service in ANC might be one mechanism to improve the quality of MNC care and increase uptake of other MNCH services in resource limited settings.

This study has strengths. First, the study used large sample size representing diverse cultural and geographic contexts, levels of facilities, and MNC service providers and users. Second, this is the first study to examine the enablers and barriers to the introduction of obstetrics ultrasound at the primary health facilities in Ethiopia. Our study also has certain limitations. First, we used data from the expanded field notes of the interviews rather than verbatim transcription of interviews. Although we provided training to the interviewers and supervisors during data collection, the data might not capture the nuances from the interviews. Second, we couldn’t analyze the data and present enablers and barriers across all health system's building blocks that might have provided a broader and better picture because our data is limited to addressing only some aspects of the health system. Third, some of the FGDs were held with few health providers, and in the hospital settings some providers interrupted the discussion to provide care to mothers, which might have affected the quality and flow of the discussion. Further studies overcoming these limitations are warranted.

## Conclusion

In conclusion, there is an enabling health system environment and client related enablers to introduce limited obstetric ultrasound services at primary health care facility level in resource limited settings. Trained, mentored, and supervised mid-level MNC providers can provide routine limited obstetric ultrasound scan service as part of the antenatal care services. This study highlights the need to address the identified barriers to maximize the uptake of the obstetric ultrasound services and sustain the use of the technology.

## Data Availability

All data analyzed in this study (codes and expanded field notes) can be made available through the corresponding author if required.

## Abbreviations

ANC: Antenatal Care
CPD: Cephalic Pelvic Disproportions
FGD: Focus Group Discussion
GEHC: General Electrical Health Care
IDI: In-depth Interview
IUFD: Intra Uterine Fetal Death
MCH: Maternal Child Health
MNC: Maternal Newborn Care
SNNP: South Nation National Peoples
WHO: World Health Organization
